# Parenting Stress and Parent Support Among Mothers With High and Low Education

**DOI:** 10.1037/fam0000129

**Published:** 2015-07-20

**Authors:** Alison Parkes, Helen Sweeting, Daniel Wight

**Affiliations:** 1 MRC/CSO Social and Public Health Sciences Unit, University of Glasgow

**Keywords:** parenting stress, parent support, grandparent

## Abstract

Current theorizing and evidence suggest that parenting stress might be greater among parents from both low and high socioeconomic positions (SEP) compared with those from intermediate levels because of material hardship among parents of low SEP and employment demands among parents of high SEP. However, little is known about how this socioeconomic variation in stress relates to the support that parents receive. This study explored whether variation in maternal parenting stress in a population sample was associated with support deficits. To obtain a clearer understanding of support deficits among mothers of high and low education, we distinguished subgroups according to mothers’ migrant and single-parent status. Participants were 5,865 mothers from the Growing Up in Scotland Study, who were interviewed when their children were 10 months old. Parenting stress was greater among mothers with either high or low education than among mothers with intermediate education, although it was highest for those with low education. Support deficits accounted for around 50% of higher stress among high- and low-educated groups. Less frequent grandparent contact mediated parenting stress among both high- and low-educated mothers, particularly migrants. Aside from this common feature, different aspects of support were relevant for high- compared with low-educated mothers. For high-educated mothers, reliance on formal childcare and less frequent support from friends mediated higher stress. Among low-educated mothers, smaller grandparent and friend networks and barriers to professional parent support mediated higher stress. Implications of differing support deficits are discussed.

Parenting stress (the stress associated with raising a child) makes optimal parenting more difficult (Dix, 1991; Deater-Deckard, 1998), and has negative consequences for children’s development (Pesonen et al., 2008; Cappa et al., 2011). Current theorizing and evidence suggest that parenting stress might be greater among parents from both low and high socioeconomic positions (SEP) compared with those from intermediate levels, but that this U-shaped distribution might be generated by different types of stressors in the two groups. Models emphasizing the key role of economic resources for children’s development suggest material hardship may lead to strained family relations, including parenting stress (Conger & Donnellan, 2007), and a recent U.S. national population study (Raphael et al., 2010) found greater parenting stress among low-income and low-educated groups of parents. In contrast, models of job demands and stress related to higher status point to the costs of higher SEP in terms of greater intrusion of employment into home life (Schieman et al., 2009). Highly educated parents may also find it less easy to adjust to the new parental role, following greater investment in a career (Nomaguchi & Brown, 2011). Strains related to employment might therefore produce higher parenting stress among high SEP groups.

Although stressors relating to lack of material resources and the demands of employment and career may combine to produce a U-shaped socioeconomic distribution of parenting stress (Figure 1a), the role of functional and emotional support for parents is missing from these current perspectives. Deficits in support for parents may be seen as representing “the other side of the coin” to a model based on stressors (Figure 1b). Additional stressors among high- and low-SEP groups create extra, although different, support needs when compared with intermediate SEP groups. The increased burden of needs among high- and low-SEP groups may in turn produce support deficits, leading to greater parenting stress. Support has commonly been conceived as having both a direct, as well as a buffering, or moderating, effect on parenting stress, with empirical evidence supporting both models among parents of young children (Ostberg & Hagekull, 2000). In the first (direct effect) model, we would expect support to have an independent positive effect in reducing parenting stress, regardless of context. In the second (buffering) model, we would expect the effects of context and support to interact. Here, support might produce greater reductions in parenting stress for parents at one end of the SEP spectrum. Neither model allows for the likely association between contextual factors and the support available to parents. A third model, which also emerged from a study of families with young children, conceives support as an intermediary (mediator), lying on the causal pathway between contextual stressors and psychological outcomes such as parenting stress (Quittner et al., 1990). Here, contextual factors are associated with the availability of support, which in turn influences parenting stress. Our study adopts this mediation model (Figure 1c) to suggest that contextual factors relating to limited informal, or social, support (from sources such as family and friends) and formal support (from sources such as childcare providers and health professionals) might contribute toward an understanding of socioeconomic differences in parenting stress.[Fig-anchor fig1]

Current evidence on SEP-variation in support deficits already points to reduced support among low SEP families (Turney & Harknett, 2010), although evidence that support is associated with lower parenting stress among low-income families with young children is mixed (Jackson, 1998; Raikes & Thompson, 2005; Chang & Fine, 2007). The role of support in mediating associations between low income and parenting stress has not (so far as we are aware) been investigated directly in a general population, although a study of families with school-age children at risk of behavior problems found that limited social support mediated links between low income and parental depression, which in turn was linked to less effective parenting (Lee et al., 2009). We know little about whether support deficits contribute to parenting stress among high SEP parents. However, because both partners in high SEP families are more likely than low SEP parents to resume full-time employment careers after the birth of a child, many high SEP parents will be unable to use a full-time working partner for childcare. This may be combined with perceived inadequate access to formal childcare; in the United Kingdom, 30% of high income families (> £45,000, around $71,000) reported insufficient local childcare places in 2011 (Ipsos MORI and Department for Education, 2013), and levels of employer-subsidized childcare are low (Hein & Cassirer, 2010). It therefore seems that some support deficits, particularly related to childcare, could lead to elevated parenting stress levels among high SEP families.

The main research aim of this study is to understand whether limited support helps to explain parenting stress among high- and low-SEP groups. In examining different support pathways for these groups, it will be helpful to consider the particular needs of migrants and single parents. There is evidence from population studies of US families with children of all ages (Raphael et al., 2010), and Swedish families with infants (Sepa et al., 2004), that parenting stress is greater in both migrants and single parents, and indications that these different population groups are associated with unique support deficits (see further below). In the United Kingdom, migrants and single parents are unequally distributed across SEP, constituting sizable population subgroups within parents of either high or low SEP. Single parenthood is most prevalent among low SEP groups (Brady & Burroway, 2012), but labor shortages have led to disproportionate immigration of workers at both ends of the SEP spectrum (Rienzo, 2013).

We continue by reviewing the literature on sources of parent support and parenting stress, focusing on support from grandparents, friends, and health professionals, and then we consider the literature on access to support according to single-parent and migrant status. We conclude with an outline of the current study and research hypotheses.

## Sources of Parent Support and Parenting Stress

Grandparents are likely to be the main source of support for many parents, providing emotional as well as financial and instrumental assistance (Thoits, 2011). Despite shifts toward greater provision of formal childcare in the United Kingdom since the 1998 National Childcare Strategy, almost two thirds of grandparents provide some form of childcare, with grandmothers playing a larger role than grandfathers (Wellard, 2011; Ipsos MORI and Department for Education, 2013). Nonetheless, surprisingly little is known about the impact of grandparent support on maternal health aside from studies of high-risk populations that cover school-age children. A study of children with Down syndrome found intergenerational contact and co-residence may be a source of tension and conflict (Hastings et al., 2002). A study of families of a child with a developmental disability found effects of grandparent support on maternal stress varied according to the type of support received, and which grandparent was responsible (Trute, 2003), but studies of general populations are lacking.

Contact with other sources of support, such as friends, may also reduce parenting stress. In part, this may derive from childcare and other instrumental support, although stress reduction is also likely to come from other parents with similar experiences providing empathy and advice, as well as modeling coping strategies (Thoits, 2011). A study of families of a child with autism spectrum disorder suggests the importance of both the quality and number of social relationships for mothers’ parenting stress (Benson, 2012), but again studies of general populations appear lacking.

Effects of professional sources of parenting support on parenting stress also appear absent from general population studies. Although a study of families of teenagers with severe intellectual disabilities found that though informal sources of support were associated with greater parental well-being, use of a wide range of professional support services was not (White & Hastings, 2004).

## Support Deficits Associated With Single-Parenthood and Migrant Status

Single parenthood has been associated with lower access to emotional support, over and above the effects of poverty, in a high-risk US sample (Harknett & Hartnett, 2011). This could partly reflect deficiencies in support from the nonresident partner (Kalil et al., 2005), but also conflict in relationships with other family members (Manji et al., 2005) and smaller support networks from family and friends (Cairney et al., 2003; Attree, 2005). In the United Kingdom, low-income groups and single parents rely much more on grandparents for childcare than do those from more advantaged backgrounds (Rutter & Evans, 2011). However, separation of the child’s parents weakens ties with paternal grandparents (Uhlenberg & Hammill, 1998), resulting in a matrifocal bias to remaining grandparent contact (Lussier et al., 2002). If a single mother is herself the child of a single parent, contact with the maternal grandfather may also be missing (McLanahan & Booth, 1989). A restricted grandparent network might result in lower overall support, whereas greater dependency on maternal grandparents might produce strains (Greenfield, 2011). Despite these likely limitations to informal support, single mothers from low SEP groups may be least likely to access professional help, being unaware of what is available, feeling professionals would not really be able to help, and fearful of interference and stigma (Attree, 2005). We know little about whether these likely differences in access to support are associated with higher levels of parenting stress found among single parents with young children (Sepa et al., 2004).

Migrants may experience difficulties in maintaining regular contact with other family members, which are in turn associated with lower perceived informal support (Uhlenberg & Hammill, 1998; Turney & Kao, 2009). In the United Kingdom, immigrant parents are less likely to be able to use grandparents for childcare as nonimmigrant parents (Rutter & Evans, 2011). Access to professional support might also be compromised by language and cultural barriers (Earner, 2007). As with single parents, it is not yet clear whether these likely support deficits are associated with higher levels of parenting stress found among migrants with young children (Sepa et al., 2004).

## The Current Study

Our study begins by examining associations between SEP and parenting stress among mothers of 10-month-old children from a nationally representative birth cohort in Scotland, United Kingdom. We use maternal education level as an indicator of SEP. Although correlated with other SEP indicators, such as household income or occupational class, education has the advantages of relative stability and of including never-employed mothers. In addition, we expect high maternal education to be more strongly related to a mother’s own employment- and career-related demands compared with partner or family SEP indicators (Nomaguchi & Brown, 2011). These considerations lead us to expect that high maternal education will be a stronger predictor of high parenting stress than other SEP indicators.

As for the United Kingdom as a whole, low SEP groups in Scotland contain a relatively high share of lone parents (Scottish Government, 2014). Both high and low SEP groups in Scotland include relatively high shares of overseas migrants (Office of the Chief Researcher & Office of the Chief Economic Adviser, 2010), and high SEP groups contain a relatively high share of internal migrants from the rest of the United Kingdom (McCollum, 2011). Because the support needs of migrants and lone parents are likely to embody unique features, and since these structural characteristics are likely to characterize sizable population subgroups among high- and low-SEP parents, we subdivide high- and low-educated mothers according to these additional structural factors. Subdivision permits us to explore both shared and different features of support for migrants at either end of the SEP spectrum, as well as ascertain the extent to which migrants’ needs resemble those of nonmigrant high- and low-SEP groups. Among low SEP groups, it will permit us to explore possible differences between the special needs of lone parents and those of migrants. Although ideally we would want to examine all combinations of migrant and lone parent status within both high- and low-educated groups of mothers, in practice subgroup size is a dominant consideration.

*Hypothesis 1:* Maternal parenting stress will differ significantly according to SEP as represented by educational qualifications, and will differ according to single-parenthood and migrant status.

We expect high- and low-educated mothers to be more likely to report high parenting stress, compared with groups with intermediate educational qualifications. We also expect migrant and single-parent status to predict parenting stress.

*Hypothesis 2:* The unavailability of support will help explain high levels of parenting stress for mothers with particular combinations of educational level and migrant status, single-parent status, or both.

We expect low-educated mothers, particularly single parents, to have smaller social networks and perceive more barriers to professional support. We expect high-educated mothers’ greater needs for childcare while they are at work to cause particular difficulties for migrants to Scotland, because of geographical distance from other family members. However, migrants among both high- and low-educated groups may all have difficulty accessing informal support not specifically related to childcare, such as emotional support.

## Method

### Data Set

Data were from the second birth cohort of the Growing Up in Scotland study, a nationally representative cohort of families with children born between 1st March, 2010 and 28th February, 2011 (ScotCen Social Research, 2013). The named study population was derived from child benefit records (a universal benefit with a 97% take up, at the time of the survey). Home interviews with the child’s primary caregiver were carried out in 2011–2012, when the child was 10 months old, by trained researchers using Computer Assisted Personal Interviewing. Data collection was subject to medical ethical review by the Ethics Committee of the School of Social and Political Sciences at the University of Edinburgh. Of all eligible families identified, *N* = 6,127 (64%) completed an interview. The analysis data set was limited to 5865 cases where the child’s natural mother provided information (exclusions *N* = 127, 2% of families contacted) and the child was a singleton birth (further exclusions *N* = 135, 2% of families contacted).

### Measures

*Maternal parenting stress* was self-reported using an abbreviated version of the Parental Stress Scale (Berry & Jones, 1995). Mothers indicated agreement with three items: “Having a child leaves little time and flexibility in my life,” “It is difficult to balance different responsibilities because of my child,” “Having a child has meant having too few choices and too little control over my life.” Responses used a 5-point scale from (1) “strongly agree” to (5) “strongly disagree”. Factor analysis indicated items loaded on one factor accounting for 57% of the variance (loadings 0.7–0.8). Mean scores (Cronbach’s alpha = .62) were used here, after reverse-coding the scale so that high scores denoted higher stress.

*Maternal education* was classified using the Scottish Credit and Qualifications framework (http://www.sqa.org.uk/sqa/4596.557.html) as (a) degree-level academic and vocational qualifications, (b) Scottish Highers and upper level vocational qualifications, (c) upper level Scottish Standard grades and intermediate level vocational qualifications, (d) lower level Scottish Standard grades and vocational qualifications and (e) no qualifications. Group 2 qualifications are those required for university entry, whereas Groups 3 and 4 represent qualifications typically attained by the minimum school leaving age. Initial exploration indicated no differences in parenting stress levels between Groups 2 and 3, and between Groups 4 and 5. These pairs were therefore combined to give a three-part classification of educational level: high, intermediate and low. Three quarters (76%) of high-educated mothers were in professional or managerial occupational classes, and 70% were in households categorized as being in the top 40% of equivalized income (> £26,000, around $41,000). In contrast, most low-educated mothers were employed in semiroutine or routine occupations (56%) or had never worked (24%), and 79% were in households with an income in the bottom 40% (< £17,000, around $27,000). *Single-parent status* was derived from questions about whether the mother had a partner, and whether the partner resided in the household. *Migrant status* was derived from a question on place of birth. Responses were coded as: Scotland; rest of United Kingdom (England, Wales, Northern Ireland) and outside the United Kingdom.

#### Support measures

*Grandparent network size* was based on mothers’ reports of the number of grandparents she was in regular contact with (either face-to-face, or by phone, letter or e-mail). For the purposes of this survey, the term “grandparent” was interpreted broadly to include nonblood relatives of the child, such as a mother’s stepfather, and so a child could be listed as having more than four “grandparents”. To prevent nontraditional family forms from dominating the network size measure, all grandparent networks greater than four were recoded as four. Further information about grandparent support was collected in respect of each individual grandparent that the mother was in touch with, and responses were aggregated to create the two following measures of support. *Grandparent contact frequency* was a scale based on how often grandparents saw the child, looked after the child for an hour or more during the day, and babysat in the evening (Cronbach’s alpha = .84), selecting the most frequent contact with any grandparent for each item. Responses were on a six-point scale (“every day or almost every day” to “never”). *Grandparent support level* was based on five types of support in the past year: taking the child on outings, buying toys, clothes or equipment, helping at home, helping financially in some other way, advice and support (yes/no response for each item). Scores counted any provision of each type of support by grandparents on either side of the family, and these were combined to give a total support measure (range 0–10). This was strongly correlated with grandparent network size (*r* = .47).

*Friend network size* was measured by asking: “How many close friends would you say you have?” with responses on a 5-point scale (“none” to “ten or more”). *Supportive friendships* were measured using the statement “My friends take notice of my opinions” (responses from 1 “strongly agree” to 5 “strongly disagree,” with response 6 “I don’t have any friends” coded as 5). *Friend contact frequency* was measured by asking: “How often do you usually see or speak to your close friends either in person, by phone, on e-mail or using the internet?” with responses on a seven-point scale (“every day or almost every day” to “less than once every three months”). Childcare use was based on questions regarding the length of care per week for each provider currently used. Hours were classified as either informal (e.g., grandparents, friend), or formal (e.g., registered child minder, nursery). *Reliance on formal childcare* was the share of total childcare hours spent with a formal provider (rescaled from 0 to 1). *Barriers to professional parent support* was a standardized scale based on five items (α = .69), indicating agreement using a 5-point scale with statements relating to interference from professionals, inadequacy of support available, stigma associated with support, lack of trust, and lack of knowledge about who to ask.

Although the main interest of the study was in support mediators of parenting stress, additional maternal and family characteristics provided information on SEP-related stressors and likely support needs. *Mother’s age* is known to be positively associated with feeling more restricted and less fulfilled by the parental role, independent of other influences (Nomaguchi & Brown, 2011); and may also be related to lower availability of maternal grandparent support, due to declining health (Wellard, 2011). Degree-educated mothers are most likely to be in full-time employment (Jaumotte, 2004) and affected by pressure from nonstandard full-time schedules (Joshi & Bogen, 2007), whereas low-educated groups may suffer more financial pressure. We assessed *employment pattern* based on hours worked at the time of interview, as well as nonstandard hours (regularly working evenings or nights). *Household money worries* were based on two items, *r* = .51, concerning difficulty repaying debts, and how well the family managed financially. We also expect low maternal health to be associated with greater parenting stress (Cornish et al., 2006; Anderson, 2008). *Maternal mental and physical health* were both measured using the SF-12 scale (Ware et al., 1996). We include a measure of family size (number of children under 16 years at interview), since this has also been linked to greater stress and lower support (Ostberg & Hagekull, 2000). Lastly, we include mother’s ethnicity (white/minority status) and language spoken at home, which may indicate acculturation difficulties (Raphael et al., 2010).

### Data Analysis

Initial bivariate analyses were performed using Stata/*SE* version 12.1 (StataCorp LP, College Station, TX). Multivariate analyses were performed using Mplus version 7.3 (Muthén & Muthén, 1998–2012). Missing data were handled using Full Information Maximum Likelihood. Analyses allowed for the complex sampling design and survey weights to correct for lower take-up of the survey among disadvantaged groups and younger mothers. There were four stages to the analysis. First, associations between maternal educational level, migrant and single-parent status and maternal parenting stress were investigated using multiple linear regression. We then subdivided high- and low-educated groups, based on important combinations of migrant and lone parent status, and compared support in these subgroups to the reference group, mothers with intermediate education. Next, we constructed path models to explore mediators of parenting stress for high- and low-educated groups. Most mediation analysis is confined to continuous or binary predictors, but recent work has shown how it is possible to test mediation using a general linear approach where the predictor is (as in this study) a multicategorical variable (Hayes & Preacher, 2014). The “model indirect” command was used to estimate pathways from maternal stress group to parenting stress. For a multicategorical predictor, this command produces “relative” indirect effects where effect size is compared with that in a specified reference group (here, mothers with intermediate education). This analysis allowed us to establish which aspects of support were important for high- and low-educated groups. A final stage modeled the effects of adjusting for mediators of parenting stress in stages to demonstrate effects of sets of mediators important for high- and for low-educated groups separately.

## Results

Just over a third of mothers (35%) were in the high-educated group with degree-level academic or vocational qualifications, 13% were in the low-educated group with low-level or no qualifications, whereas the remaining 52% had intermediate qualifications. Table 1 shows the uneven distribution of migrant status and single-parent status across these three educational groups. Around three in 10 high-educated mothers and two in 10 mothers with low education were born outside Scotland, compared with 12% of mothers with intermediate education. Nearly half of mothers with low education lacked a resident partner, compared with only 4% of degree-educated mothers. Table 2 shows that all three maternal characteristics were associated with parenting stress, even when mutually adjusted. Stress was higher in mothers with high *and* low education (compared with intermediate), those born outside Scotland, particularly outside the United Kingdom (compared with in Scotland) and single mothers (compared with those with a partner). No interaction term between factors was significant (not shown), indicating additive rather than multiplicative effects. Although the reference group used here for maternal education is the intermediate group, resetting the reference group as high education indicated highest stress among low-educated mothers in the unadjusted model (coefficient 0.10, *p* = .03), reduced to a nonsignificant difference (0.06, *p* = .08) after adjusting for migrant and lone parent status. [Table-anchor tbl1][Table-anchor tbl2]

To explore support deficits among high- and low-educated groups of mothers, we then disaggregated these two groups further. Among high-educated mothers, external and internal migrants were separated from native born mothers. Among low-educated mothers, external migrants in couple families and lone parents (regardless of country of birth) were separated from U.K.-born (i.e., native Scottish born and internal migrant) couple families. This procedure gave a total of six “maternal stress groups,” together with the intermediate-educated reference group.^1^

The maternal stress subgroups all had higher parenting stress than the reference group (first line of Table 3). A supplementary table S1 shows differences in maternal and characteristics for the seven groups. Both high- and low-educated groups contained relatively high shares of mothers who were from a minority ethnic group, or whose families did not speak any English at home. However, there were also differences in how high- and low-educated groups compared with the intermediate-educated reference group. High-educated groups were older, more likely to be in full-time employment, and more likely to work full-time nonstandard hours. Low-educated groups were all less likely to be working; and (with the notable exception of low-educated external migrants) had poorer health, more money worries and larger families. Overall, these contrasts suggest the possibility of some similarities, but also substantial differences, in support needs at either end of the maternal education spectrum. Similarities are likely to reflect the presence of migrants at both ends. Differences may reflect the greater need for regular childcare when the mother is at work among high-educated groups, but greater need for multiple forms of support (financial and emotional, as well as instrumental) among low-educated groups.[Table-anchor tbl3]

These considerations seem to be borne out by patterns of support shown in the main part of Table 3. Less frequent grandparent contact was evident for high-educated and the low-educated groups containing migrants, since comparatively few had grandparents residing in the local area. However, less frequent contact was also seen among low-educated couples born in the United Kingdom, even though most had grandparents nearby. Expected differences in support were also apparent at either end of the spectrum. All high-educated groups were more reliant on formal childcare and enjoyed less regular contact with friends, compared with the intermediate group. At the low-educated end of the spectrum, grandparent and friend network size and support levels were reduced compared with the intermediate-educated reference group, and there were more perceived barriers to professional parent support. A small grandparent network was particularly evident among low-educated single mothers: further analysis found 51% had no contact with paternal grandparents, and 21% were restricted to contact with one grandparent only (typically the maternal grandmother). These figures compare with 19% and 7%, respectively, in the whole sample.

Path models were constructed to test the mediating effects of support measures for parenting stress. Model 1 examined mediators for the six maternal stress groups, relative to the intermediate reference group, whereas Model 2 compared the high and low maternal education groups with the intermediate reference. Indirect (mediating) effects are shown in Table 4. Bold figures indicating significant positive effects (i.e., a group’s *higher* stress is mediated by a given support measure), whereas italicized figures indicate significant negative effects (i.e., a group’s *lower* parenting stress is mediated via a given support measure). For Model 1, with the exception of grandparent contact frequency (mediating higher parenting stress for two high-educated and two low-educated groups), there were different sets of mediators of higher stress for high-educated mothers and for low-educated mothers. Reliance on formal childcare and lower friend contact frequency mediated higher stress among high-educated groups, whereas smaller grandparent and friend network size and perceived barriers to professional parent support mediated higher parenting stress among low-educated groups. It is also striking to note that mediators of greater parenting stress among low-educated mothers were also often significant pathways to *lower* stress (italicized figures) among high-educated mothers, and vice versa. These effects were also observed in the simpler Model 2. Sensitivity analyses were conducted to explore the effect of varying the mediator measures and controlling for maternal mental health, which may have biased perceptions of support and stress. Substituting grandparent support level and friends’ supportiveness measures for network size measures found similar effects, suggesting network size equated to lower overall support. Controlling for maternal mental health to allow for possible bias to reports of parenting stress and support did not alter the findings (supplemental file S2).[Table-anchor tbl4]

Lastly, we explored the effect of adding mediators shown to be relevant for particular groups to models of associations between maternal education and parenting stress. Table 5 shows that adjusting for grandparent contact frequency reduced coefficients for both high-educated and low-educated mothers, with the effect for low-educated external migrants in couple families becoming nonsignificant (Stages 1 and 2 compared). Further adjustment for reliance on formal childcare and friend contact frequency (Stage 3) produced further reductions for high-educated mothers, with effects for internal migrants and native born groups becoming nonsignificant. Effects for low-educated mothers were largely unchanged from Stage 2. Alternative further adjustment for grandparent and friend network size and barriers to informal support (Stage 4) reduced coefficients for low-educated groups, to nonsignificance for UK-born couple families; although effects for high-educated groups increased rather than decreased. In Model 2, Stages 3 and 4 adjustments for support deficits, respectively, reduced the effect of high and low education on parenting stress by around 50% in both groups.[Table-anchor tbl5]

## Discussion

This study found higher maternal parenting stress among both most and least educated mothers of infant children compared with mothers with intermediate education. As far as we know, ours is the first study to document higher stress among high- as well as low-educated groups, and the first to explore how this socioeconomic variation in stress relates to maternal support. To obtain a clearer understanding of support deficits among high-and low-educated groups, we subdivided them according to mothers’ migrant and single-parent status. These two additional structural factors were unequally distributed among high- and low-educated groups, with a high share of migrants among high- and low-educated mothers, and a high share of lone parents among low-educated mothers. Migrant and single-parent status were also associated with higher parenting stress, as found for another large population study of families with infant children immigrant status (Sepa et al., 2004). Collectively, these findings regarding inequalities in parenting stress support our first hypothesis.

Overall, support deficits accounted for around half of the higher parenting stress experienced by high- and low-educated mothers, supporting our second hypothesis. We found that less frequent grandparent contact helped to explain higher parenting stress among both high- and low-educated mothers, with this effect found predominantly among migrants. Aside from this, degree-educated and low-educated mothers appeared to lack different dimensions of informal support. Stress among low-educated mothers was associated with smaller and less effective networks. Stress among high-educated mothers was associated with less readily accessible informal support, despite larger network size and quality. Reliance on formal childcare was a particular source of stress for high-educated mothers, who were more likely to be in full-time employment than less-educated groups. Barriers to professional support were most pertinent for low-educated mothers.

Existing studies suggest the importance of grandparents for mothers’ parenting stress in families where children have a particular health problem or disability (Hastings et al., 2002; Trute, 2003), and the current study underscores the importance of grandparent contact in providing childcare and more general support to mothers within a general population. Our data suggest lower availability of maternal grandparent support was limited by geographical distance for migrants. Weaker family ties also appeared pertinent for reduced contact among some disadvantaged groups, as found in other research on low-income families with young children (Harknett & Hartnett, 2011). Even after accounting for regular childcare arrangements, less frequent grandparent contact was associated with greater parenting stress among high-educated migrants; perhaps reflecting less regular emotional, as well as functional, support. Among less-educated groups, smaller grandparent networks appeared more relevant. This echoes other research finding smaller grandparent networks reduce financial as well as emotional support (Harknett & Hartnett, 2011). For low-educated single-parent mothers, there were indications that support restricted to the maternal grandmother was a particularly strong mediator of parenting stress. This might seem counterintuitive, since a closer bond between a mother and maternal grandparents has been well established in the literature (Chan & Elder, 2000). In part, exclusion of paternal grandparents might be a choice made by mothers faced with more challenges, to avoid interference or criticism (Harknett & Hartnett, 2011). However, overdependence on the maternal grandmother may put stress on this relationship (Greenfield, 2011).

Differences between degree- and low-educated groups in support from friends resembled differences in support from grandparents. Stress among low- educated mothers appeared related to lower emotional support from friends, in keeping with research suggesting social relations may deteriorate when a mother is unable to reciprocate support (Harknett & Hartnett, 2011); though degree-educated mothers’ higher stress was associated with less frequent contact with friends, including by remote means. This may reflect longer working hours and reduced leisure time. In showing independent effects of grandparents and friends on parenting stress, our findings extend previous research using combined support measures, and tend to support the idea that empathy and shared experiences involving other parents of young children provide an extra dimension to maternal support beyond that obtained from an older generation of relatives (Thoits, 2011). Indeed, a study of adolescent mothers with infant children suggested that friends may be particularly important for younger mothers, outweighing the importance of family support for parenting stress (Richardson et al., 1995). Lastly, mistrust and low awareness of formal support among disadvantaged mothers with infant and preschool children has been documented in a study of an earlier Scottish cohort (Mabelis & Marryat, 2011). Our study suggests that these barriers to professional support helped to explain higher levels of parenting stress for mothers with low education, even after allowing for differences in maternal mental health that might produce a negative bias to perceptions.

Strengths of the study include the nationally representative population sample, and the presence of detailed information on different sources of support, especially contact with grandparents. However, it also has several potential limitations. It relied on mothers for sensitive information, lacked information on fathers’ stress, used abbreviated scales for some measures (including parenting stress), and some previously unvalidated measures. Maternal education was used as an indicator of SEP, although analyses using household income or area deprivation produced a similar U-shaped distribution of parenting stress (Parkes et al., 2013). Migrant status was defined by country of birth since we lacked information on length of residence in Scotland. Some migrants might have moved to Scotland as children with their parents, and have similar access to them as locally born respondents. Numbers did not permit further disaggregation of groups according to partner migrant status. Two additional important limitations are the cross-sectional study design, coupled with the possibility of bias in mothers’ reports of support. It is possible that high parenting stress may have biased attitudes to professional support, but we adjusted for poor mental health and our use of contact-based (rather than perceived) informal support measures is likely to have countered bias here. Lastly, it is important to note that causal processes cannot be inferred from this type of study. It is possible that omitted variables might be responsible for some of the associations found, and might help account for remaining high stress, especially among some groups of mothers (high-educated external migrants, and low-educated single parents). Other research has related partner support to maternal parenting stress (Kalil et al., 2005; Mulsow et al., 2002; Nomaguchi & Brown, 2011), but we lacked sufficient information to model this. Acculturation difficulties are likely to contribute to external migrants’ perceptions of stress and need for support (Raphael et al., 2010). Although low-educated external migrants’ stress appeared to be driven by grandparent contact frequency, acculturation difficulties might contribute to stress among high-educated external migrants striving to combine a career with caring for an infant child.

Overall, our study suggests that understanding socioeconomic variation in parenting stress levels may be enhanced by further disaggregating the population to highlight particular groups at risk from low support. Disaggregation demonstrates the need for different approaches to mitigate contextual factors associated with parenting stress among different population groups, thus going some way toward informing more effective interventions (Pawson & Tilley, 1997). In Scotland, there is a rapidly growing immigrant population (Krausova & Vargas-Silva, 2013), and this study adds to concerns for immigrant parents’ integration into informal support networks (Turney & Kao, 2009). High parenting stress was also found for internal migrants from the rest of the United Kingdom, which was also related to childcare problems among mothers returning to work, and more generally to a lack of informal support. This is likely to reflect the geographical remoteness of Scotland from the rest of the United Kingdom, and further research is needed to establish whether internal migrants elsewhere experience similar difficulties. There is evidence to suggest the beneficial effects of Internet and telephone resources for new mothers’ well-being (McDaniel et al., 2012). However, although remote social connections may provide some emotional support, lack of functional support may pose more problems and this study adds to concerns about the ability of formal childcare provision to meet childcare needs (Wheelock & Jones, 2002). For the most disadvantaged families, an Irish qualitative study (Doyle et al., 2010) suggests benefits to children from reconnection with estranged grandparents. It seems important to establish whether there are also benefits to the parent from repaired grandparent relationships. In addition, our study raises questions over the use of professional support to compensate for family support deficits. It adds to qualitative evidence that sensitivity is required to overcome feelings of mistrust or stigma attached to the use of professional services among disadvantaged mothers with young children (Attree, 2005), although there is now evidence of the effectiveness of some types of group intervention in reducing maternal distress (Barlow et al., 2012). Finally, although we found the highest levels of parenting stress among the most disadvantaged mothers, our findings point to the need to avoid assumptions that parenting stress is uniquely associated with disadvantage and to expand efforts beyond women living in poverty with limited educational resources. Our study underlines the desirability of taking a more sophisticated approach to risk assessment. Despite some similarity between high-and low-educated migrants’ needs, in most respects high- and low-educated groups had opposite sets of support needs. Indeed, a support deficit at one end of the SEP spectrum was seen to constitute a relative support advantage at the other end of the spectrum. In this way, high-educated mothers’ larger informal networks and low perceived barriers to professional parent support alleviated their parenting stress compared with intermediate mothers, whereas having accessible informal childcare was a source of lower parenting stress among low-educated groups. This suggests a prime need for tailored outreach and targeted interventions to maximize benefits to different groups of mothers, as well as efficient use of resources.

## Supplementary Material

10.1037/fam0000129.supp

## Figures and Tables

**Table 1 tbl1:** Maternal Migrant and Partner Status According to Level of Maternal Education

Maternal characteristic	High education (*n* = 2, 129)		Intermediate education (*n* = 2, 898)		Low education (*n* = 691)		Total sample (*N* = 5, 717)	
	*n*	%	*n*	%	*n*	%	*n*	%
Mother’s country of origin								
Scotland	1,495	70.5	2,523	87.7	540	79.3	4,558	80.6
Rest of UK	346	15.7	274	9	61	8.2	681	11.2
Outside of UK	288	13.8	100	3.4	90	12.4	478	8.2
Resident partner status and origin								
Scotland	1,457	68.1	1,836	61.8	286	39.6	3,579	61
Rest of UK	350	16.1	238	7.6	30	4	618	10.1
Outside of UK	238	11.6	114	3.9	75	10.3	427	7.4
No partner	82	4.2	709	26.7	300	46.1	1091	21.5
*Note.* Percentages relate to columns and take account of the complex survey design. UK = United Kingdom.								

**Table 2 tbl2:** Associations Between Maternal Characteristics and Parenting Stress

Maternal characteristic	Reference	β	
		Stage 1–Unadjusted	Stage 2–Mutually adjusted
Education	Intermediate		
	High	0.10***	0.10***
	Low	0.20***	0.17***
Migrant status	Scotland		
	Rest of UK	0.07*	0.07*
	Outside UK	0.22***	0.17***
Partner status	Yes (reference)		
	No	0.10***	0.12***
*Note.* Interactions between maternal characteristics were not statistically significant. UK = United Kingdom.			
* *p* < .05. ** *p* < .01. *** *p* < .001.			

**Table 3 tbl3:** Means and Standard Errors (in Parentheses) Parenting Stress and Support Among Mothers Grouped According to Education, Migrant, and Single-Parent Status

			Maternal groups based on education level (high/intermediate/low), migrant, and single-parent status							Comparisons	
		Total sample	1. High-overseas migrant	2. High-internal migrant	3. High-native	4. Intermediate (reference)	5. Low-native couple	6. Low-overseas couple	7. Low-single parent	High vs. ref.^a^	Low vs. ref.^a^
Stress and support measures	*N*	5,717	288	346	1,495	2,897	313	78	300		
Parenting stress	Higher	2.61 (0.01)	2.82 (0.04)	2.70 (0.03)	2.59 (0.02)	2.54 (0.01)	2.67 (0.04)	2.76 (0.08)	2.80 (0.05)	1, 2, 3	5, 6, 7
Support for parenting											
Grandparent(s) live in local area	Yes	84.3	33.7	47.2	83.3	92.8	92.3	29.4	93.7	1, 2, 3	6
Grandparent in household	Yes	6.2	3.3	2.1	2.2	7.3	3.7	5.5	20.8	1, 2, 3	5, 7
Use regular childcare	Yes	53.2	45.0	54.7	60.8	54.3	40.6	18.1	38.2	1, 3	5, 6, 7
Reliance on formal childcare^b^	Increasing	0.17 (0.01)	0.28 (0.03)	0.33 (0.03)	0.23 (0.01)	0.14 (0.01)	0.08 (0.01)	0.08 (0.03)	0.06 (0.01)	1, 2, 3	5, 7
Grandparent network size	Increasing	3.08 (0.02)	3.14 (0.05)	3.42 (0.05)	3.36 (0.02)	3.06 (0.02)	2.69 (0.06)	2.72 (0.14)	2.27 (0.06)	2, 3	5, 6, 7
Grandparent support level	Increasing	5.21 (0.04)	4.72 (0.17)	5.48 (0.12)	5.75 (0.06)	5.29 (0.05)	4.47 (0.15)	2.93 (0.37)	4.29 (0.13)	1, 3	5, 6, 7
Grandparent contact frequency	Decreasing	−0.47 (0.01)	0.18 (0.05)	−0.19 (0.05)	−0.54 (0.02)	–0.57 (0.01)	–0.41 (0.05)	0.41 (0.10)	–0.53 (0.05)	1, 2	5, 6
Friend network size	Increasing	3.16 (0.02)	3.18 (0.06)	3.54 (0.05)	3.47 (0.03)	3.08 (0.02)	2.90 (0.06)	2.61 (0.12)	2.75 (0.05)	2, 3	5, 6, 7
Supportive friendships	Increasing	4.14 (0.01)	4.12 (0.04)	4.25 (0.04)	4.31 (0.02)	4.13 (0.01)	3.93 (0.05)	3.84 (0.12)	3.88 (0.06)	2, 3	5, 6, 7
Friend contact frequency	Decreasing	2.15 (0.02)	2.50 (0.08)	2.49 (0.07)	2.45 (0.03)	2.01 (0.02)	2.02 (0.07)	2.17 (0.13)	1.75 (0.06)	1, 2, 3	7
Barriers to professional support	Increasing	2.40 (0.01)	2.35 (0.04)	2.21 (0.03)	2.22 (0.02)	2.43 (0.01)	2.67 (0.04)	2.66 (0.08)	2.74 (0.05)	2, 3	5, 6, 7
*Note.* ref. = reference group, mothers with intermediate education.											
^a^ Values in this column refer to group number(s) showing a significant difference (*p* < .05) with Reference Group 4 (mothers with intermediate education). ^b^ Estimates include mothers not using regular childcare.											

**Table 4 tbl4:** Indirect Effects of Maternal Characteristics on Parenting Stress via Support Mediators

	Support mediators					
Model main predictors	Grandparent network size	Grandparent contact frequency	Reliance on formal childcare	Friends network Size	Friend contact frequency	Barriers to professional parent support
Model 1						
Maternal group by status						
High-educated, external migrant	−0.001	0.016***	0.005**	−0.001	0.008***	−0.005
High-educated, internal migrant	−0.004***	0.008***	0.006***	–0.005**	0.009***	–0.016***
High-educated, native	−0.006***	0.001	0.006***	–0.009**	0.015***	–0.030***
Low-educated, UK-born couple	0.004**	0.004**	–0.002**	0.002*	0.000	0.018***
Low-educated, external migrant couple	0.002*	0.012***	–0.001	0.003*	0.002	0.009**
Low-educated, single parent	0.009***	0.001	–0.003**	0.004**	–0.005**	0.024***
Model 2						
Maternal group by education						
High-educated	−0.007***	0.009***	0.008***	−0.010**	0.017***	−0.030***
Low-educated	0.009***	0.007***	−0.003***	0.005**	−0.002	0.030***
*Note.* Reference group in both models consist of mothers with intermediate education. Figures show standardized estimates. Values in bold indicate significant positive indirect effects (i.e. mediation of higher parenting stress in maternal group via support measure). Values in italics indicate significant negative indirect effects (i.e. mediation of lower parenting stress in maternal group via support measure). UK = United Kingdom.						
* *p* < .05. ** *p* < .01. *** *p* < .001.						

**Table 5 tbl5:** Associations Between Maternal Groups and Parenting Stress After Adjustment for Support Mediators

			β			
Model main predictors			Stage 1-Unadjusted	Stage 2-Adjusted for migrant support deficit	Stage 3-Adjusted for high-educated support deficits	Stage 4-Adjusted for low-educated support deficits
Model 1						
Maternal group based on education, migrant and lone parent status	High-educated, external migrant		0.29***	0.20***	0.17***	0.25***
	High-educated, internal migrant		0.16***	0.12**	0.08	0.21***
	High-educated, native		0.05*	0.05*	0.01	0.13***
	Low-educated, UK-born couple		0.13***	0.12**	0.12**	0.05
	Low-educated, external migrant couple		0.22**	0.11	0.12	0.05
	Low-educated, single parent		0.26***	0.26***	0.28***	0.15**
Support mediators	Grandparent network size	Larger				−0.03***
	Grandparent contact frequency	Decreasing		0.12***	0.10***	0.09***
	Reliance on formal childcare	Greater			0.08**	
	Friends network size	Larger				−0.05***
	Friend contact frequency	Decreasing			0.06***	
	Barriers to professional parent support	Greater				0.14***
Model 2						
Maternal group based on education only	High-educated		0.10***	0.08***	0.05*	0.16***
	Low-educated		0.20***	0.18***	0.19***	0.09**
Support mediators	Grandparent network size	Larger				−0.03***
	Grandparent contact frequency	Decreasing		0.12***	0.11***	0.09***
	Reliance on formal childcare	Greater			0.08**	
	Friends network size	Larger				−0.05***
	Friend contact frequency	Decreasing			0.06***	
	Barriers to professional parent support	Greater				0.14***
*Note.* Reference group in both models: mothers with intermediate education. Values show nonstandardized coefficients.						
* *p* < .05. ** *p* < .01. *** *p* < .001.						

**Figure 1 fig1:**
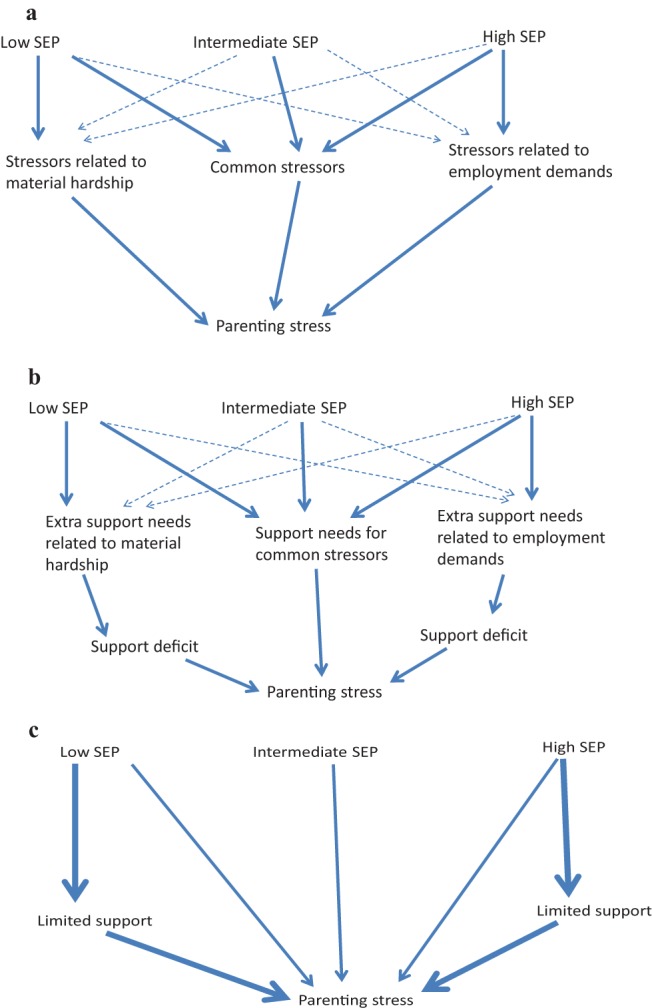
Conceptual models of associations between parental socioeconomic position (SEP) and parenting stress: (a) stressor model, (b) support needs/deficits model, and (c) support limitation model. All models hypothesize additional pathways to parenting stress for high- and low-SEP groups, compared with the intermediate group: This produces higher stress among high- and low-SEP groups. Models (a) and (b): Solid arrows indicate strong positive associations; dashed arrows indicate relatively weak or absent associations. Model (c): Bold arrows indicate the main pathways of interest in this study, suggesting limited support mediates associations between high-or low-SEP and parenting stress. See the online article for the color version of this figure.

## Footnotes

1 Note that there were insufficient numbers to create separate groups for high-educated migrant lone parents (*N* = 23), high-educated native lone parents (*N* = 59), low-educated internal migrants (*N* = 41), or low-educated migrant lone parents (*N* = 32).
